# PMMS: Predicting essential miRNAs based on multi-head self-attention mechanism and sequences

**DOI:** 10.3389/fmed.2022.1015278

**Published:** 2022-11-17

**Authors:** Cheng Yan, Changsong Ding, Guihua Duan

**Affiliations:** ^1^School of Informatics, Hunan University of Chinese Medicine, Changsha, China; ^2^School of Computer and Information, Qiannan Normal University for Nationalities, Duyun, China; ^3^School of Computer Science and Engineering, Central South University, Changsha, China

**Keywords:** microRNA, essential miRNA, bi-directional long short-term memory, multi-head self-attention mechanism, deep learning

## Abstract

Increasing evidence has proved that miRNA plays a significant role in biological progress. In order to understand the etiology and mechanisms of various diseases, it is necessary to identify the essential miRNAs. However, it is time-consuming and expensive to identify essential miRNAs by using traditional biological experiments. It is critical to develop computational methods to predict potential essential miRNAs. In this study, we provided a new computational method (called PMMS) to identify essential miRNAs by using multi-head self-attention and sequences. First, PMMS computes the statistic and structure features and extracts the static feature by concatenating them. Second, PMMS extracts the deep learning original feature (BiLSTM-based feature) by using bi-directional long short-term memory (BiLSTM) and pre-miRNA sequences. In addition, we further obtained the multi-head self-attention feature (MS-based feature) based on BiLSTM-based feature and multi-head self-attention mechanism. By considering the importance of the subsequence of pre-miRNA to the static feature of miRNA, we obtained the deep learning final feature (WA-based feature) based on the weighted attention mechanism. Finally, we concatenated WA-based feature and static feature as an input to the multilayer perceptron) model to predict essential miRNAs. We conducted five-fold cross-validation to evaluate the prediction performance of PMMS. The areas under the ROC curves (AUC), the F1-score, and accuracy (ACC) are used as performance metrics. From the experimental results, PMMS obtained best prediction performances (AUC: 0.9556, F1-score: 0.9030, and ACC: 0.9097). It also outperformed other compared methods. The experimental results also illustrated that PMMS is an effective method to identify essential miRNA.

## Introduction

MicroRNAs (miRNAs) are a class of typical non-coding RNAs (ncRNAs) of 22 nucleotide (nt) in length, express endogenously, and regulate gene expression on the posttranscriptional level. During miRNA biogenesis, Drosha and Dicer process the primary transcript (pri-miRNA) through a precursor hairpin (pre-miRNA) to the mature miRNA (1). In other words, RNA polymerase II transcribes the nuclear genes of miRNA to generate pri-miRNAs. The pre-miRNA is produced from pri-miRNAs based on the hairpin structure with enzymatic cleavage. miRNAs often interact with 3' untranslated region (3'UTR) of a target mRNA to mediate mRNA degradation and/or translational repression (2). Furthermore, some studies also demonstrated that they also interact with other regions, such as 5'UTR, gene promoters, and coding sequence (3).

The first two known miRNAs, namely lin-4 and let-7, were derived from *Caenorhabditis elegans* and were discovered more than 20 years ago (4, 5). Until recently, thousands of currently annotated miRNAs have been identified in a variety of species from plants and animals to viruses (6, 7). miRNAs can be released into the extracellular environment and transported to the target cells by vesicles, including exosomes, or *via* binding to proteins. Once expressed, miRNAs are integrated into the RISC and guide the repression of a target mRNA by base complementarity within the “seed” sequence of the miRNA (8). This process resulted in either repression or degradation of the target mRNA and affected the differentiation and proliferation of cells. In addition, many pieces of evidence have proven that miRNAs play essential roles in some important biological processes, such as cell growth, proliferation (9), differentiation (10), and development (11).

Furthermore, many studies demonstrated that miRNAs are highly correlated with human complex diseases, and essential miRNA is crucial in animal development and human diseases. For example, miR-144-3p was lowly expressed in non-small cell lung cancer (NSCLC) and might function as a potential tumor biomarker in the prognosis prediction for NSCLC (12). miR-200c-141 and miR-200b-200a-429 were downregulated in human breast cancer stem cell (BCSC), normal human and murine mammary stem/progenitor cells, and embryonal carcinoma cells (13). miR-200c inhibited the clonal expansion of breast cancer cells and suppressed the growth of embryonal carcinoma cells *in vitro*. In colorectal cancer (CRC), miRNAs-21 is one of the most important miRNAs and is also emerging as a biomarker in CRC, with good potential as a diagnostic and therapeutic target (14). miR-125a-5p could be considered a regulator of glycolipid metabolism in type 2 diabetes mellitus (T2DM), which can inhibit hepatic lipogenesis and gluconeogenesis and elevate glycogen synthesis by targeting STAT3 (15). The expression of miR-145 is significantly downregulated in dedifferentiated vascular smooth muscle cells (VSMCs) and in balloon-injured arteries, which can be considered a potential therapeutic target (16). miR-228 was also upregulated in osteoarthritis (OA), and can be considered a biomarker (17). In addition, after knocking out miR-15b, B cell lymphoproliferative disorders in mice have been observed (18). After knocking out miR-144, the incidence of spontaneous B lymphoma and acute myeloid leukemia in aged mice increased (19).

Due to the importance mentioned earlier and the necessity of miRNA-disease associations and essential miRNAs, a growing number of databases have been developed. miRbase was an online repository for nomenclature and annotation, and the latest release (v22) contains microRNA sequences from 271 organisms: 38,589 hairpin precursors and 48,860 mature microRNAs (20). miRGator was also a miRNA portal for deep sequencing, expression profiling, and mRNA targeting (21). In addition, many miRNA-disease association databases have also been established, which include miR2Disease (22), human microRNA disease database (HMDD) (23, 24), ExcellmiRDB (25), and miRCancer (26). miR2Disease was a manually curated database and represented an exhaustive resource of miRNA deregulation in different human diseases. HMDD was also an miRNA-disease association database and was developed in 2007, and the HMDD v3.2 gathered more than 35,547 experimentally confirmed entries of miRNA-disease association containing about 1,206 miRNA genes and 893 diseases from 19,280 papers. ExcellmiRDB was also a user-friendly and curated online database about miRNA-disease associations, which includes 1,108 extracellular miRNAs-biofluid relationships and 2,773 extracellular miRNA-disease derived from 108 papers selected from >600 PubMed abstracts. miRCancer collected 878 associations between 236 miRNAs and 79 human cancers through the processing of >26,000 published articles. Besides, Cui et al. (27) established an essential miRNA benchmark dataset to predict potential essential miRNAs.

Due to the importance and necessity of miRNA and the development of the miRNA-disease association database and essential miRNA benchmark dataset, a growing number of computational methods have been proposed for the prediction of miRNA-disease association and essential miRNA. For example, BNPMDA was a typical network-based method to predict potential miRNA-disease associations by using the known miRNA-disease association network, integrated miRNA similarity network, and integrated disease similarity network (28). DNRLMF-MDA was a miRNA-disease association prediction method based on dynamic neighborhood regularized logistic matrix factorization. The main feature of DNRLMF-MDA was that known miRNA-disease associations are assigned higher importance levels than unknown miRNA-disease associations (29). Chen et al. also proposed a method to predict new miRNA-disease association by completing the missing miRNA-disease association based on the known associations and the integrated miRNA similarity and disease similarity (30). Based on the matrix decomposition and heterogeneous graph inference model, MDHGI was proposed to predict potential miRNA-disease associations (31). It improved the prediction accuracy (ACC) by taking full advantage of matrix decomposition before the construction of a heterogeneous network. Zhou et al. also proposed a method of neural inductive matrix completion with the graph convolutional network (NIMCGCN) for identifying miRNA-disease association (32). NIMCGCN first extracted the latent feature representations and disease from the miRNA and disease similarity networks. PDMDA was also an miRNA-disease association prediction method based on the graph neural network (33). It can predict not only miRNA-disease association but also predict the association type. miES was the first essential miRNA prediction method by using pre-miRNA and miRNA sequences and it also constructed the benchmark dataset of essential miRNA (27). PESM was also an essential miRNA prediction method and improved the prediction performance by adding new features and gradient boosting machines model (34). In addition, XGEM was also an essential miRNAs prediction method by applying the XGBoost framework with Classification and Regression Trees (CART) on various types of sequence-based features (35).

Although we have obtained some progress in predicting essential miRNA based on the development of computing technology and essential miRNA benchmark dataset, it is critical to propose new computational method to improve the prediction ACC. In this study, we provided a method (predicting essential miRNAs based on the multi-head self-attention and sequences, PMMS) to predict essential miRNA. PMMS first calculates the static feature based on the pre-miRNA and miRNA sequences by statistics and the Vienna RNA Package (34). The deep learning original feature (BiLSTM-based feature) is obtained by bi-directional Long Short-Term Memory (BiLSTM) and pre-miRNA sequences. The multi-head self-attention feature (MS-based feature) of miRNA is extracted based on multi-head self-attention mechanism and BiLSTM-based feature. The deep learning final feature (WA-based feature) of miRNA is obtained by using a weight attention mechanism with a static structure feature and MS-based feature of miRNA. Finally, we obtained the final feature of miRNA by concatenating the static feature and WA-based feature, and then takefeature, and then taken it as input into Multilayer perceptron (MLP) to predict essential miRNA. We conducted five-fold cross-validation to evaluate the prediction performance of PMMS and compared it with other computational methods which includes PESM, miES, GaussianNaiveBayes (Gaus NB), and support vector machines (SVM) models. The the areas under the ROC curves (AUC), F1-score, and ACC are used as metrics. The experimental results showed that PMMS obtains the best prediction performance according to AUC value of 0.9556. The ACC (0.9037) and F1-score (0.9030) of PMMS in the five-fold cross-validation were higher than that of other methods, respectively, and it also proved that it can achieve better results.

## Materials

In this study, we also used the benchmark dataset of essential miRNA which was also used in PESM and miES. This benchmark dataset includes 77 known essential miRNAs that were confirmed by knocking out gene experiments. It also includes the same number of negative samples which are randomly selected from the unknown essential miRNAs. In addition, by considering the production process of mature miRNA and the hairpin structure of pre-miRNA, we also used the pre-miRNA and miRNA sequences which are downloaded from the miRbase database. miRbase provided the nomenclature and annotation and pre-miRNA sequences and mature-miRNA sequences of humans, rats, and mice. The latest release (v22) contains miRNA sequences from 271 organisms: 38,589 hairpin precursors and 48,860 mature miRNAs.

## Methods

**Figure 1:**
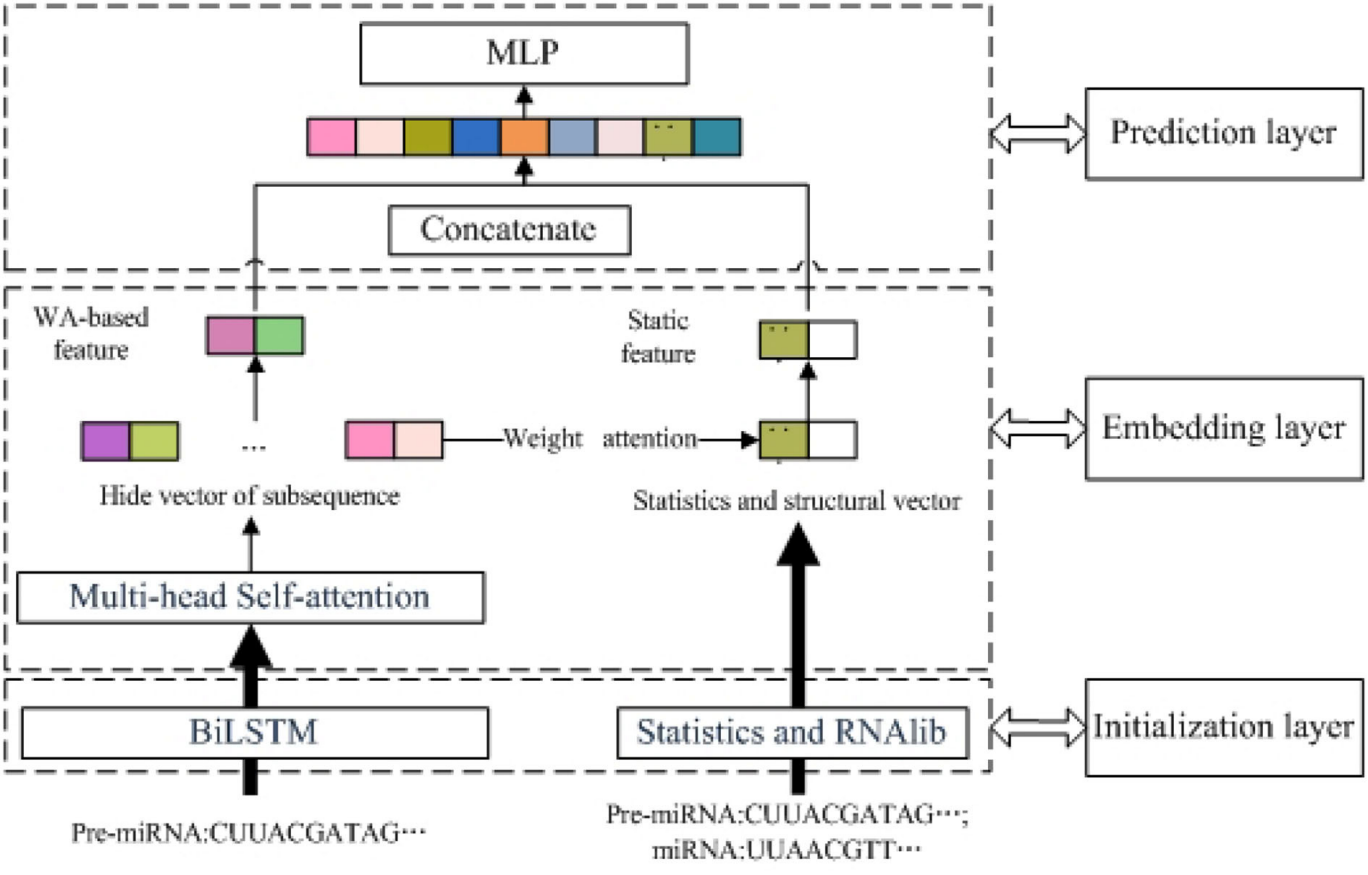
The overview of predicting essential miRNAs based on the multi-head self-attention and sequences (PMMS) approach.

As shown in Figure 1, PMMS mainly contains three layers. The left section of the initialization layer extracted the BiLSTM-based feature by k-mer and the BiLSTM model. The right section of the initialization layer obtained the static feature (statistic and structure feature) by calculating the number of nucleotides and RNAlib package. In addition, we obtained the MS-based feature through the multi-head self-attention mechanism and BiLSTM-based feature. In the embedding layer, we also obtained the WA-based feature based on the MS-based feature and weight attention mechanism. Finally, the miRNA final feature is obtained by concatenating the WA-based feature and static feature and is input into the MLP model to predict essential miRNA. The identification of essential miRNA is also a typical binary-classification problem, and MLP has also been successfully applied to the data classification problem.

### Statistic and structure feature

Based on the production process of mature miRNA, we calculated the statistic and structure feature of miRNA from pre-miRNA sequence and mature miRNA sequence. We all know that RNA polymerase II transcribes the nuclear genes of miRNA to generate pri-miRNAs which produced pre-miRNA by enzymatic cleavage on the hairpin structure. Then, the mature miRNA is produced through cleaving pre-miRNA. Since the mature miRNA sequence is the subsequence of the pre-miRNA sequence, we let non-mature miRNA denote the rest of the pre-miRNA sequence after cleaving miRNAs. First, we calculated the single nucleotide base content *S* ∈ {*U, C, G*} in pre-miRNA, miRNA, and non-mature miRNA sequences. Three features with dimensionality 3 were obtained, respectively. The sequence lengths of pre-miRNA and miRNA sequences were also calculated as the feature with dimensionality 1, respectively. In addition, we also calculated the number of dinucleotide pairs *S, Z* ∈ {*U, C, G*} in pre-miRNA and miRNA sequences. Thus, we obtained features with dimensionality 9 from them, respectively. The cleavage site base class is another feature and is divided into three categories: (1) 1 represents all cleavage sites of mature-miRNAs from the same pre-miRNAs are *U*; (2) 0 represents not all cleavage sites are *U*; (3) −1 represents all are *non* − *U*. Based on the hairpin structure contained in all pre-miRNAs which can produce mature miRNA, we also calculated the structure feature by the Vienna RNA Package. The minimum free energy (MFE) and nMFE (minimum free energy and it is divided by its length) are the feature with dimensionality 1, respectively. In addition, we also further considered the base-pairing propensity, Shannon entropy, and base-pair distance to obtain 6 features which include normalized base-pairing propensity (*dP*), normalized base-pairing propensity divided by its length (*dP*/*L*), normalized Shannon entropy (*dQ*), normalized Shannon entropy divided by its length (*dQ*/*L*), normalized base-pair distance (*dD*), and normalized base-pair distance divided by its length (*dD*/*L*). These features were widely used in miRNA prediction (36) and pre-miRNA prediction (37). Table 1 describes the overview of all statistics and structure features.

**Table 1:** The overview of statistics and structure feature *F*_*s*_.

**Category**	**Description**	**Dimensionality**
Base content in pre-miRNAs	The content *S* in pre-miRNA, *S* ∈ *U, C, G*	3
Base content in miRNA	The content *S* in mature-miRNA, *S* ∈ *U, C, G*	3
Base content in non-miRNA	The content *S* in non-mature-miRNA, *S* ∈ *U, C, G*	3
miRNA length	The sequence length of mature-miRNAs	1
non-mature miRNA length	The sequence length of non-mature miRNAs	1
Cleavage site base class	The cleave sites are assigned into 3 classes, 1: all cleavage sites of mature-miRNAs from the same pre-miRNAs are *U*; 0: not all cleavage sites are *U*; − 1: all are *non* − *U*	1
Dinucleotide pairs number in pre-miRNA	The Dinucleotide pairs *SZ* number in pre-miRNAs, *S, Z* ∈ *U, C, G*	9
Dinucleotide pairs number in miRNA	The Dinucleotide pairs *SZ* number in mature-miRNAs, *S, Z* ∈ *U, C, G*	9
MFE and nMFE	The minimum free energy of pre-miRNA secondary structures and it is divided by its length	2
The base-paring structure feature of pre-miRNAs	normalized base-pairing propensity (*dP*), normalized base-pairing propensity divided by its length (*dP*/*L*), normalized Shannon entropy (*dQ*), normalized Shannon entropy divided its length (*dQ*/*L*), normalized base-pair distance (*dD*), normalized base-pair distance divided by its length (*dD*/*L*)	6

### BiLSTM-based feature

By considering the successful application of BiLSTM in natural language processing (NLP) (38) and the timing characteristic of pre-miRNA sequence, we also applied BiLSTM to extract the deep learning original feature. Compared with the LSTM model (39, 40), BiLSTM was provided to encode information back to front when using LSTM to model the sequences. It includes two LSTMs that are used to take the input in a forward direction and a backward direction. For pre-miRNA sequences, BiLSTM can not only process sequences in temporal order but also consider the future context. LSTM is composed of a cell, an input gate, an output gate, and a forget gate. The cell remembers values over arbitrary time intervals. The three gates regulate the flow of information into and out of the cell. Based on the design characteristics of LSTM and the characteristic of time series data, LSTM is very suitable for processing text and biological sequence data.

To apply the BiLSTM to pre-miRNAs, we first also defined “word” in pre-miRNA sequences as k-mer nucleotide. There are 4 types of NNs (A, U, C, and G). In this study, we set *k* to be 3 based on the experiment results. Therefore, we can split a pre-miRNA sequence into an overlapping 3-mer nucleotide. For pre-miRNA “AUUGUCC...”, the 3-mer nucleotide is defined as follows:


(1) $$\begin{array}{l} “AUU^{\prime \prime },“UUG^{\prime \prime },“UGU^{\prime \prime },“GUC^{\prime \prime },“UCC^{\prime \prime },... \end{array}$$

After obtaining the 3-mer nucleotide of pre-miRNA sequences, we translated them to randomly initialized embeddings (word embedding). For a pre-miRNA sequence *S* = *s*_1_, *s*_2_, *s*_3_, ..., *s*_|*s*|−1_, *s*_|*s*|_, the 3-mer embedding of pre-miRNA sequence can be concatenated with nucleotide embeddings, and it is defined as follows:


(2) $$\begin{array}{l} \left[s_{1},s_{2},s_{3}\right],\left[s_{2},s_{3},s_{4}\right],...,\left[s_{|S|-1},s_{|S|-2},s_{|S|-3}\right], \end{array}$$

where *x*_*i*_ = [*s*_*i*_; *s*_*i*+1_; *s*_*i*+2_] and *d*_*b*_ = 128. After obtaining 3-mer embedding of sequence, we take it as input to the BiLSTM model.

**Figure 2:**
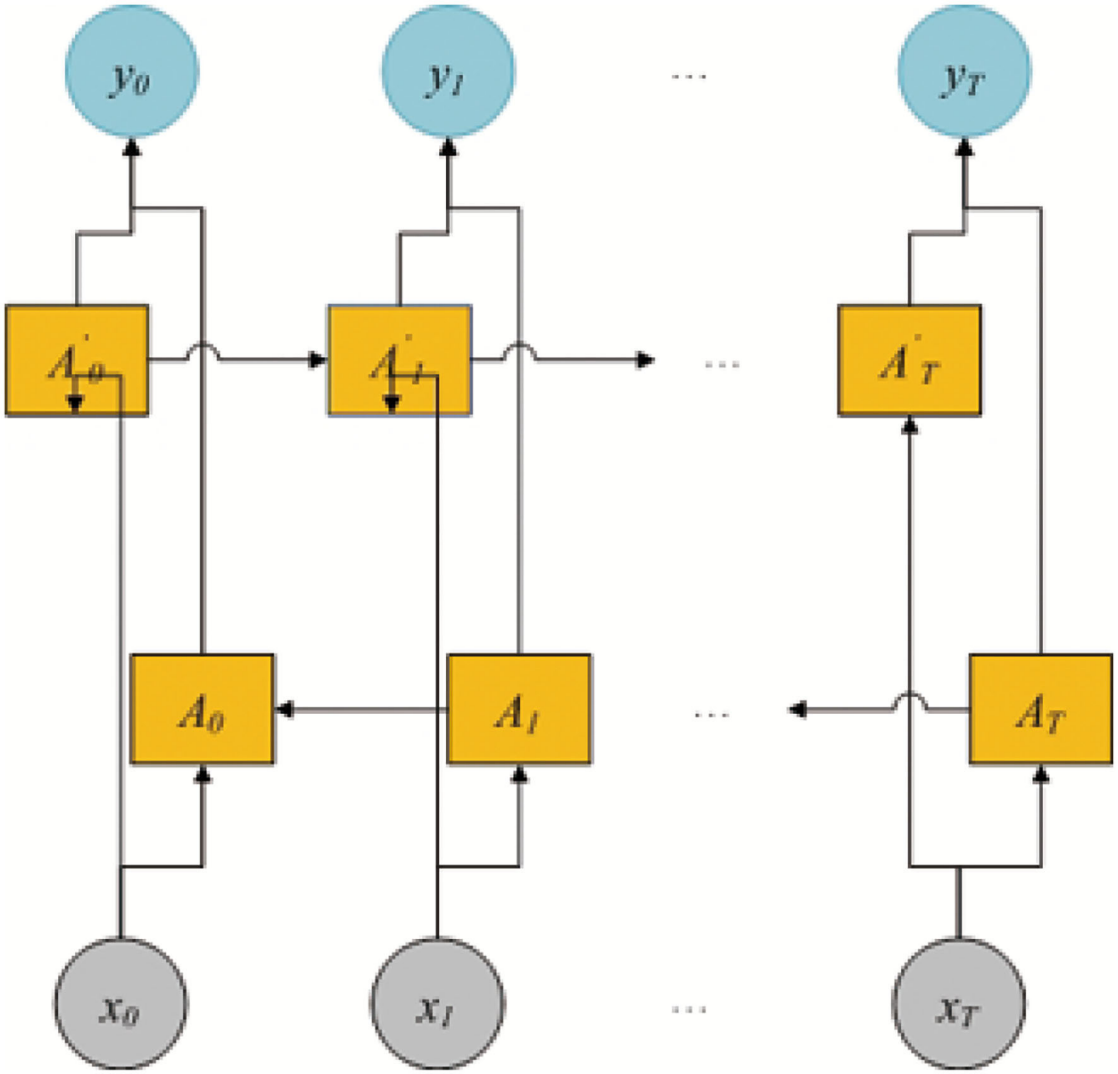
The overview of the bi-directional long short-term memory model.

The BiLSTM model is shown in Figure 2. The model includes two LSTM sub-networks for the left and right nucleotide sequenceright nucleotide sequences. They are forward and backward passes, respectively. The final output of the i-th 3-mer nucleotide sequence is computed by using an element-wise sum of forward and backward pass outputs. In this study, the input of BiLSTM is the 3-mer embedding of pre-miRNA sequence $F_{B0}^{\left(t\right)}\in R^{d_{b}}=x_{i}$ and *d*_*b*_ = 128. The output of BiLSTM is the BiLSTM-based feature $F_{B}=\left\{F_{B1}^{\left(t\right)},F_{B2}^{\left(t\right)},...,F_{B|L|}^{\left(t\right)}\right\}$, |*L*| = |*S*| − 2, and $F_{Bi}=y_{i}\in R^{d_{b}}$.

### MS-based feature

By considering the application of multi-head self-attention mechanism (41) in learning tasks with contextual relationships which include drug-target interaction (42–44), prediction, etc. The multi-head self-attention mechanism can address the limits that LSTM cannot obtain long-dependent information when the sequence is long.

**Figure 3:**
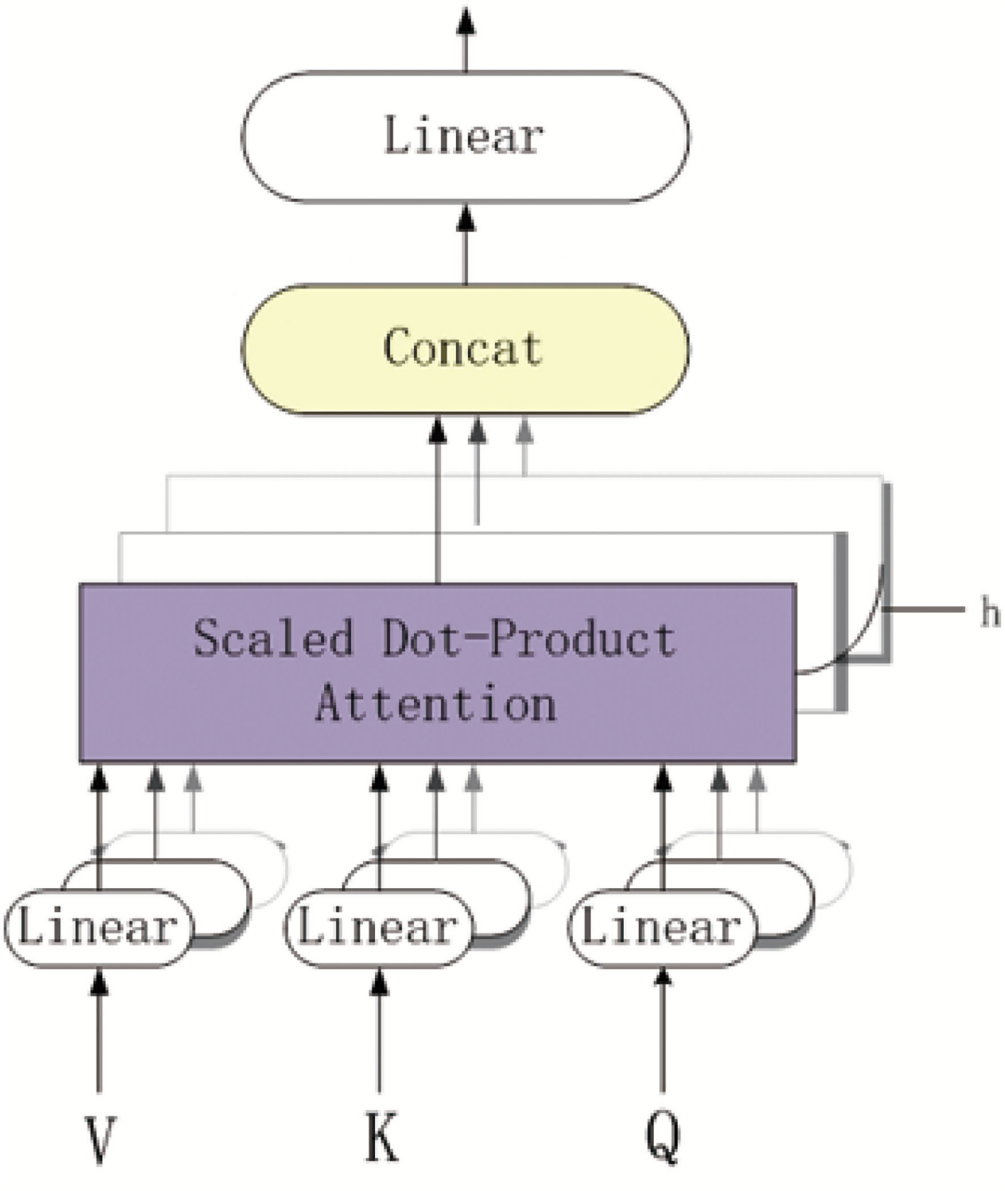
The overview of the multi-head self-attention model.

Figure 3 shows the overview of the multi-head self-attention mechanism model. The part of purple background block is the scaled dot-product attention model. As depicted in Figure 3, each k-mer nucleotide vector in a pre-miRNA sequence can be represented as a query(Q) and key(K)-value(V) pair by the three mapping matrices. The parameters of these matrices are learned by backward propagation. The output of each k-mer nucleotide can be obtained by mapping a query and a set of KV pairs to get the weighted sum at different locations in the pre-miRNA sequence.

More specifically, after obtaining the BiLSTM-based feature of pre-miRNA, we can get the representation of Q and K-V matrices of each k-mer nucleotide. The dimensionality of Q and K is *d*_*k*_, and the dimensionality of V is *d*_*v*_. We also computed the dot products of the Q with all K, dividing each by $\sqrt{d_{k}}$, and applied a softmax function to obtain the weights on the values. Based on the queries, keys and values are packed into matrices Q, K, and V, the output matrix of the scaled dot-product attention model is as follows:


(3) $$\begin{array}{l} Attention\left(Q,K,V\right)=softmax\left(\frac{Qk^{T}}{\sqrt{d_{k}}}\right)V \end{array}$$

In addition, the multi-head attention mechanism can jointly attend to information from different representation subspaces at different positions. For the output multi-head attention mechanism, the computation process is defined as follows:


(4) $$\begin{array}{l} MultiHead(Q,K,V)=Concat(head_{1},...,head_{h})W^{o},\\ \;where\;head_{i}=Attention(QW_{i}^{Q},KW_{i}^{K},VW_{i}^{V}), \end{array}$$

in which the projections are mapping matrices $W_{i}^{Q}\in R^{d_{input}*d_{k}}$, $W_{i}^{K}\in R^{d_{input}*d_{k}}$, $W_{i}^{V}\in R^{d_{input}*d_{v}}$, and $W_{o}\in R^{hd_{v}*d_{output}}$. The parameters setting are *d*_*input*_ = *d*_*b*_ = 128, *d*_*k*_ = *d*_*v*_ = 64, *d*_*output*_ = 38 and *h* = 4 The matrices Q, K, and V are initialized by the BiLSTM-based feature *F*_*B*_. Therefore, after above process, we obtained the MS-based feature *F*_*M*_ = {*F*_*M*1_, *F*_*M*2_, ..., *F*_*M*|*L*|_} where $F_{Mi}\in R^{d_{output}}$.

### WA-based feature

After obtaining the static feature *F*_*s*_ and MS-based feature *F*_*M*_, we further consider the importance of k-mer nucleotide to static feature *F*_*s*_. We compute which k-mer nucleotide in the pre-miRNA are more important for the static feature *F*_*s*_ by assigning greater weights to this k-mer nucleotide. The detail computation process of weight attention is defined as follows:


(5) $$\begin{array}{l} h_{m}=f(w_{inter}F_{s}+b_{inter}),\\ h_{i}=f(w_{conv}F_{Mi}^{\left(t\right)}+b_{inter}),\\ \alpha_{i}=\sigma (h_{m}^{T}h_{i}), \end{array}$$

in which *W*_*inter*_ and *b*_*inter*_ are the weight matrix and bias vector, respectively. *f* is the rectified linear unit (ReLU) active function. Attention matrix *W*_*inter*_ represents the importance between statistic and structure feature *F*_*s*_ and k-mer nucleotide of pre-miRNA. Therefore, the WA-based feature *F*_*W*_ can be calculated by the weighted sum of *h*_*i*_ with attention and is defined as follows:


(6) $$\begin{array}{l} F_{W}=\overset{L}{\underset{i=1}{\sum }}\alpha_{i}h_{i}. \end{array}$$

### Essential miRNA prediction based on MLP

The essential miRNA prediction is a typical binary-classification problem. In this study, we used the MLP model to identify essential miRNA. After obtaining the static feature *F*_*s*_ and WA-based feature *F*_*w*_, we concatenated them as the final miRNA feature $F_{f}\in R^{d_{f}}$ where *d*_*f*_ = 76. Then, we took it as input for the MLP model to predict essential miRNA. The hidden vector of t-th layer can be computed as follows:


(7) $$\begin{array}{l} h_{t}=f\left(W_{h}h_{t-1}\right)+b_{h}, \end{array}$$

in which *W*_*h*_ and *b*_*h*_ are the weight matrix and bias vector, and all learned by back propagation process. In addition, *f* is ReLU active function. Note that the input is $h_{0}=F_{f}\in R^{d_{f}}$. Finally, the output vector *z* can be computed as follows:


(8) $$\begin{array}{l} z=\left(W_{o}h_{t}\right)+b_{o}, \end{array}$$

where $W_{o}\in R^{2*d_{f}}$ and $b_{o}=R^{2}$ are the weight matrix and the bias vector, respectively. Based on the output vector *Z* = [*o*_0_, *o*_1_], the essential miRNA probability can be computed by a softmax function and it is defined as follows:


(9) $$\begin{array}{l} \begin{array}{c} p_{l}=\frac{exp\left(o_{l}\right)}{\sum_{i}o_{i}}, \end{array} \end{array}$$

where *l* ∈ {0, 1} is the label and *p*_*l*_ is the probability of label *l*. In addition, the cross-entropy loss is also used as the loss function and is defined as follows:


(10) $$\begin{array}{l} \begin{array}{c} loss_{cross}=-\frac{1}{N}\overset{N}{\underset{i}{\sum }}\overset{1}{\underset{l}{\sum }}y_{i,l}log\left(p_{i,l}\right), \end{array} \end{array}$$

where *y*_*i,l*_ and *y*_*i,l*_ are the real and predicted one-hot representation on label *l* of i-th sample, respectively. If the i-th sample belongs to label *l*, then *y*_*i,l*_ = 1, otherwise *y*_*i,l*_ = 0. *N* is the number of samples in the training dataset. Therefore, the training objective is to minimize the function *loss* and is defined as follows:


(11) $$\begin{array}{l} \begin{array}{c} loss\left(\theta \right)=-\frac{1}{N}\overset{N}{\underset{i}{\sum }}\overset{1}{\underset{l}{\sum }}y_{i,l}log\left(p_{i,l}\right)+\frac{\lambda }{2}\|\theta {\|}_{2}^{2}, \end{array} \end{array}$$

where θ is the set of all weight matrices and bias vectors. The parameter λ is the L2 regularization hyper-parameter.

## Result

### Comparison with previous methods

In this study, we conducted five-fold cross-validation (5CV) to evaluate the prediction performance of our methods and other compared methods, which include PESM (34), miES (27), Gaus_NB (34), and SVM (34). They are essential miRNA prediction methods. In addition, the AUC, ACC, and F1-score are used as metrics to measure the performance of prediction results. The higher the values of AUC, ACC, and F1-score are, the better the method performs. In 5CV, we randomly divided all samples into five subsets with equal size. Then, each subset is in turn considered a test sample, and the rest subsets are treated as training samples.

**Figure 4:**
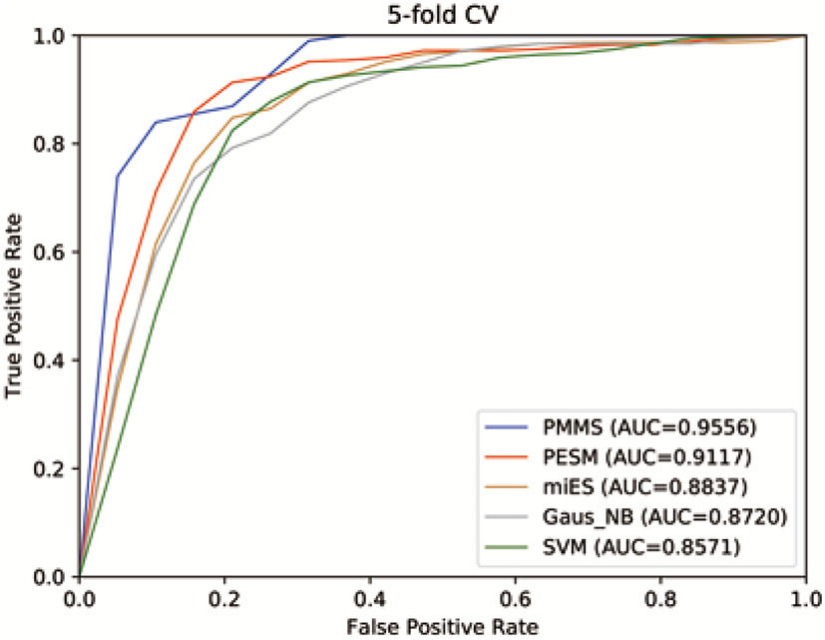
The receiver operating characteristic (ROC) curves of PMMS and other compared methods.

Figure 4 shows the receiver operating characteristics (ROC) curve and AUCs of PMMS and other compared methods. If AUC = 1, it would indicate that all test samples were perfectly predicted, while AUC = 0.5 would mean the model only had random prediction performance. We can observe from Figure 4 that PMMS obtained an AUC of 0.9556 in 5 CV. However, the AUCs of PESM, miES, Gaus_NB, and SVM are 0.9117, 0.8837, 0.8720, and 0.8571, respectively. It illustrates that our method can obtain better prediction performance than other compared methods.

Furthermore, Table 2 also demonstrates the ACC, F1-score, and AUC values of PMMS and other compared methods. PMMS obtained the ACC and F1-score values of 0.9097 and 0.9030, respectively. In addition, the best ACC and F1-score values of compared methods were 0.8516 and 0.8572, respectively. It also demonstrated that our method outperformed other compared methods according to ACC and F1-score values.

**Table 2:** The ACC, F1-score, and AUC values of five methods on the five-fold cross validation (5CV).

**Method**	**PESM**	**miES**	**Gaus_NB**	**SVM**	**PMMS**
ACC	0.8516	0.8263	0.8000	0.8206	**0.9097**
F1-score	0.8572	0.8326	0.8093	0.8271	**0.9030**
AUC	0.9117	0.8837	0.8720	0.8571	**0.9556**

The bold values represent the best performance.

### Model and parameter analysis

In this study, we also analyzed the influence of prediction performance on different parameters. Furthermore, we also analyzed the feature learning ability of our method.

The parameter *k* is used to obtain the BiLSTM-based feature in k-mer of pre-miRNA sequences. We can observe from Table 3 that PMMS achieved better prediction results when *k* is set to be 3 (AUC:0.9557) or 4 (ACC:0.9097, F-measure:0.9030) on the 5CV. Our method has stable prediction performance when *k* ranged from 3 to 6, the AUC values were 0.9494, 0.9557, 0.9556, 0.9421, and 0.9541, respectively. Therefore, we set the default value of parameter *k* to 4.

**Table 3:** The prediction performances of predicting essential miRNAs based on the multi-head self-attention and sequences (PMMS) with different settings of *k*.

***k***	**2**	**3**	**4**	**5**	**6**
ACC	0.8996	0.8966	**0.9097**	0.8837	0.8901
F1-score	0.8856	0.8927	**0.9030**	0.8791	0.8832
AUC	0.9494	**0.9557**	0.9556	0.9421	0.9541

The bold values represent the best performance.

**Figure 5:**
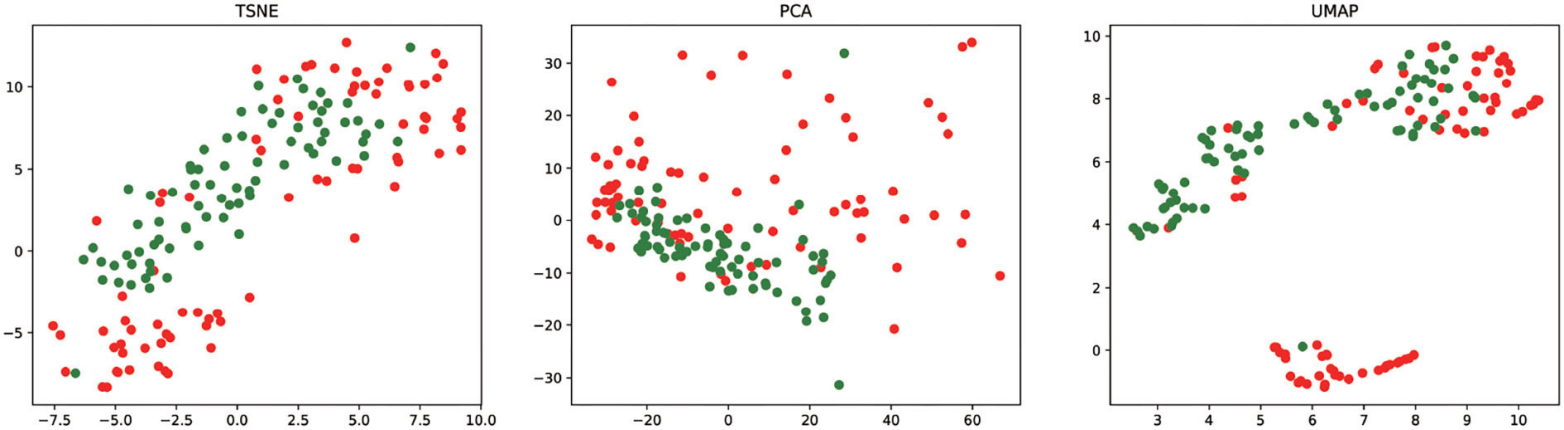
The final miRNA feature vectors of the test sets are visualized after dimensionality reduction by t-SNE, PCA, and UMAP. The red circle and green circle represent the essential miRNAs and unknown essential miRNAs, respectively.

To analyze the feature learning ability of our method, we project the feature vectors into the two-dimensional feature space and visualize the result of essential miRNA classification based on the 5CV. We take the final miRNA feature as input to t-SNE (45), PCA (46), and UMAP (47) for reducing the feature dimensionality. Figure 5 shows the visualization result of essential miRNA classification based on the final miRNA feature by reducing the feature dimensionality by three methods. We can observe from Figure 5 that PMMS can distinguish essential miRNA from unknown essential miRNA in three feature dimensionality reduction methods. In addition, it also shows that compared with t-SNE and PCA dimensionality reduction methods, UMAP is relatively more obvious in distinguishing the samples.

## Discussion

MiRNAs are an important class of single-stranded ncRNA molecules that have close association with human diseases. In addition, some miRNAs are also essential through knocking out gene experiments. Due to the importance of miRNA to human disease, it is very urgent to identify potential essential miRNAs. It is also very important to systematically understand the mechanisms of the etiology and pathogenesis of diseases. However, since identifying potential essential miRNAs *via* biomedical experiments is expensive and time-consuming, the effective computational methods for essential miRNAs prediction are in demand. Currently, some essential miRNAs prediction methods have been proposed by researchers. They also provide a basis for the development of new computational methods.

## Conclusion

In this study, we also proposed a new computational method (PMMS) to predict essential miRNAs. PMMS first calculated the statistics and structure features based on the pre-miRNA and miRNA sequences. By considering the timing characteristic of the pre-miRNA sequence, we also obtained the original deep learning feature by the BiLSTM model. The multi-head self-attention-based feature is obtained by original deep learning feature and multi-head self-attention mechanism. Furthermore, by further considering the importance of the subsequence of pre-miRNA to statistics and structure feature of miRNA, the final deep learning feature is obtained by weight attention mechanism with the statistics and structure feature and multi-head self-attention-based feature. Finally, we concatenated the statistics and structure feature and the final deep learning feature as an input to the MLP model to predict essential miRNAs. The experiment results demonstrated that our method outperformed other compared methods and is an effective essential miRNA prediction approach.

However, despite the effectiveness of PMMS as discussed above, some limits also exist in this method. The first limitation is that the number of known essential miRNAs is relatively small based on the limit of benchmark dataset. We would construct a new benchmark dataset by extracting the essential miRNAs from published literatures. In addition, other biological networks of miRNAs should be considered, such as miRNA-target associations. Furthermore, new deep learning model should also be considered based on added biological networks, such as GCN and other models (48–50). Therefore, we would develop new computational method to improve the ACC of essential miRNA prediction method in the future.

## Data availability statement

Publicly available datasets were analyzed in this study. This data can be found here: http://www.cuilab.cn/mies.

## Author contributions

CY abstracted the main ideas, conducted a comparative assessment, analyzed the results, and wrote the manuscript. CD and GD conceived the subject, instructed the experimental design, analyzed the results, and revised the manuscript. All authors contributed to the article and approved the submitted version.

## Funding

The authors would like to express their gratitude for the support from the National Natural Science Foundation of China (Nos. 61962050 and 62072473), Natural Science Foundation of Hunan Province of China (No. 2022JJ30428), Science and Technology Foundation of Guizhou Province of China under Grant No. [2020]1Y264, and Natural Science Foundation of Education of Guizhou Province (No. KY[2017]351).

## Conflict of interest

The authors declare that the research was conducted in the absence of any commercial or financial relationships that could be construed as a potential conflict of interest.

## Publisher's note

All claims expressed in this article are solely those of the authors and do not necessarily represent those of their affiliated organizations, or those of the publisher, the editors and the reviewers. Any product that may be evaluated in this article, or claim that may be made by its manufacturer, is not guaranteed or endorsed by the publisher.
